# Functional differentiation of Sec13 paralogues in the euglenozoan protists

**DOI:** 10.1098/rsob.220364

**Published:** 2023-06-14

**Authors:** Drahomíra Faktorová, Kristína Záhonová, Corinna Benz, Joel B. Dacks, Mark C. Field, Julius Lukeš

**Affiliations:** ^1^ Institute of Parasitology, Biology Centre, Czech Academy of Sciences, České Budějovice, Czech Republic; ^2^ Faculty of Sciences, University of South Bohemia, České Budějovice, Czech Republic; ^3^ Faculty of Science, Charles University, BIOCEV, Vestec, Czech Republic; ^4^ Life Science Research Centre, Department of Biology and Ecology, Faculty of Science, University of Ostrava, Ostrava, Czech Republic; ^5^ Division of Infectious Diseases, Department of Medicine, Faculty of Medicine and Dentistry, University of Alberta, Edmonton, Canada; ^6^ Centre for Life's Origins and Evolution, Department of Genetics, Evolution, and Environment, University College London, London, UK; ^7^ School of Life Sciences, University of Dundee, Dundee, UK

**Keywords:** *Diplonema*, coatomer, membrane trafficking, nuclear pore complex, SEA/GATOR complex, paralogue expansion

## Abstract

The β-propeller protein Sec13 plays roles in at least three distinct processes by virtue of being a component of the COPII endoplasmic reticulum export vesicle coat, the nuclear pore complex (NPC) and the Seh1-associated (SEA)/GATOR nutrient-sensing complex. This suggests that regulatory mechanisms coordinating these cellular activities may operate *via* Sec13. The NPC, COPII and SEA/GATOR are all ancient features of eukaryotic cells, and in the vast majority of eukaryotes, a single Sec13 gene is present. Here we report that the Euglenozoa, a lineage encompassing the diplonemid, kinetoplastid and euglenid protists, possess two Sec13 paralogues. Furthermore, based on protein interactions and localization studies we show that in diplonemids Sec13 functions are divided between the Sec13a and Sec13b paralogues. Specifically, Sec13a interacts with COPII and the NPC, while Sec13b interacts with Sec16 and components of the SEA/GATOR complex. We infer that euglenozoan Sec13a is responsible for NPC functions and canonical anterograde transport activities while Sec13b acts within nutrient and autophagy-related pathways, indicating a fundamentally distinct organization of coatomer complexes in euglenozoan flagellates.

## Graphical abstract

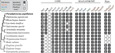

## 1. Introduction

Adaptation is essentially development or modification of novel functions and can arise through multiple mechanisms. Paralogue gene expansion is a common mechanism, which can provide new functions based on repurposing a pre-existing gene product. We, and others, have argued that paralogy gene expansion is one of several drivers that facilitated eukaryogenesis and the origins of intracellular compartments in eukaryotic cells [1–3]. While eukaryogenesis itself encompassed many events, evolution and diversification is an ongoing process, with the appearance of lineage-specific genes frequently associated with paralogue expansions [4,5]. Membrane-trafficking and related processes are examples of a highly evolvable system with many expansions and losses observed between the configurations in different organisms, likely reflecting both the central importance of these organelles and their high degree of evolutionary plasticity. The membrane-coating and deforming protocoatomer complexes that comprise most vesicle coats, intraflagellar transport and the nuclear pore complex (NPC) are ancient, with reconstructions placing the majority of complexes as already present in the last eukaryotic common ancestor (LECA) [6]. Elaboration within individual lineages, by creation of paralogues, losses of components or of entire complexes has been reported extensively, demonstrating that despite an ancient origin and central role within eukaryogenesis, considerable plasticity allows for ongoing modifications to vesicle transport and organellar complexity. Furthermore, it has been argued that the protocoatomer architecture, based around an evolvable β-propeller/α-solenoid protein is exceptionally well suited for the evolution of new functionality [7].

The COPII complex is central to anterograde membrane-trafficking from the endoplasmic reticulum (ER) to the Golgi apparatus and was present in the LECA. Canonically, it is composed of seven core subunits, of which five (Sar1, Sec13, Sec23, Sec24 and Sec31) are near ubiquitous. The two remaining subunits, Sec12 and Sec16, are less well conserved [8]. Sec12 is a guanine nucleotide exchange factor that activates the Sar1 GTPase by enhancing exchange of GDP for GTP and is located at the ER membrane. Sar1:GTP recruits the Sec23/Sec24 complex and subsequent recruitment of Sec13/Sec31 heterodimers generates the COPII coat. A previously unrecognized pan-eukaryotic paralogue of Sar1, SarB, was recently reported, which may suggest diversification within COPII recruitment mechanisms [9]. Sec16 is part of a protein scaffold, which locates to ER exit sites (ERES) and is essential for COPII recruitment, but is also implicated in non-conventional exocytosis and autophagy [10,11]. However, the absence of Sec16 from many lineages questions the generality of Sec16 essentiality in COPII transport [8].

Sec13, a β-propeller protein and a member of the extensive protocoatomer family, is a component of at least three distinct complexes, and these promiscuous interactions may reflect a deeper role in the coordination of multiple processes, *albeit* with details presently unclear. Besides a role in ER-derived transport, Sec13 is also a component of the NPC and the SEA/GATOR (for Seh1-associated/GTPase activating protein activity towards RAGA GTPase, respectively) protein complex [12–14]. Sec13 provides positive regulation to TORC1 (target of rapamycin complex 1) signalling and hence nutrient status sensing, and is instrumental in the assembly of the COPII membrane-deforming coat [15]. Sec13, COPII, the NPC and SEA/GATOR are all well conserved throughout eukaryotes, with only one Sec13 paralogue found in most lineages.

Diplonemids are highly diverse heterotrophic flagellates and very abundant in the oceans [16]. They form a sister group to the mostly parasitic kinetoplastids and represent the third arm of the Euglenozoa. Several diplonemids are bacterivorous [17], although the lifestyle of most species remains unknown, and probably ranges from phagotrophy through predation to parasitism [18]. Importantly, these ecologically and evolutionary highly relevant protists recently joined the narrow group of genetically tractable organisms [19], and gene function can now be studied in the model species *Paradiplonema papillatum* (renamed from *Diplonema papillatum*) [18,20].

As the COPII complex is well-conserved, we considered this as an excellent target for study in *P. papillatum* and selected PpSec13 (PpSec13 and TbSec13 etc. are *P. papillatum* and *Trypanosoma brucei* proteins, respectively) for analysis and to address the question of how conserved the role of Sec13 is in this divergent organism. Importantly, there are two Sec13 paralogues in kinetoplastid flagellates, and with the availability of diplonemid genomes and/or transcriptomes we asked whether the same state is present in this sister lineage. We find that Sec13 is indeed present as two paralogues, PpSec13a and PpSec13b, but unexpectedly the functionality of Sec13 in *P. papillatum* is divided, with PpSec13a involved in conventional COPII and NPC functions, while PpSec13b interacts with PpSec16 and SEA subunits and therefore participates in autophagy and nutrient-sensitive functions. Sec13 division of labour is an early feature of the whole Euglenozoa lineage, suggesting a distinct strategy for regulating the multiple functions of Sec13, and hence also multiple cellular systems.

## 2. Results

### 2.1. COPII components in diplonemids

**Figure 1.**
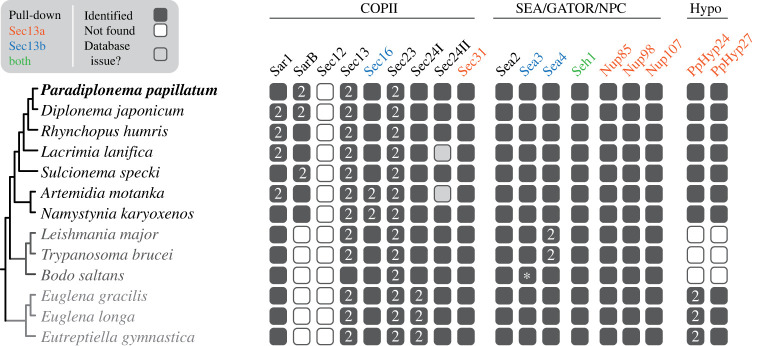
Overview of presence of COPII, SEA/GATOR/NPC and hypothetical (Hypo) subunits/proteins in Euglenozoa. Proteins pulled down with PpSec13a or PpSec13b proteins or both are shown in orange, blue or green, respectively. The annotation of sequences was confirmed by reverse best hit and/or phylogenetic analyses. The only notable exception was *B. saltans* Sea3 (marked by asterisk), which retrieved Sea4 in BLAST against *P. papillatum* proteins, but Sea3 against heterolobosean *N. gruberi* (electronic supplementary material, table S7). In the phylogenetic tree, it clustered together with Sea3 sequences from trypanosomatids, and thus we concluded that it is in fact Sea3 as well. For individual phylogenetic trees, see electronic supplementary material, figures S1 and S2.

Using sequences of COPII subunits from other members of the Euglenozoa and *Naegleria gruberi*, a representative of the Euglenozoan sister lineage Heterolobosea [8], we used homology-searching to identify genes encoding homologous proteins in the genome of *P. papillatum* [21] and recently available transcriptomes from six additional diplonemid species, namely *Diplonema japonicum*, *Rhynchopus humris*, *Lacrimia lanifica*, *Sulcionema specki*, *Artemidia motanka* and *Namystynia karyoxenos* [22]. We identified orthologues for Sar1, Sec13, Sec23, Sec24 and Sec31 (figure 1; electronic supplementary material, tables S1 and S2), whereas the less conserved Sec12 and Sec16 were not found, even with more sensitive hidden Markov model searches. Several of the conserved subunits were duplicated in all or most diplonemid species. To investigate these duplications, we performed phylogenetic analyses including sequences of relatives from the kinetoplastid and euglenid lineages.

COPII components are present in the parasitic trypanosomatids *T. brucei*, *Leptomonas pyrrhocoris* and *Leishmania major* [23–25] and the free-living euglenid *Euglena gracilis* [26]. Moreover, these components are functionally equivalent to the orthologues in animals and fungi based on localization of the respective proteins and functional evidence from genetic manipulations [27–29]. We expanded sampling by including the free-living kinetoplastid *Bodo saltans* and the euglenids *Euglena longa* and *Eutreptiella gymnastica*, to fully represent the diversity of euglenozoans. Sar1 was duplicated within the diplonemid lineage, but only one orthologue was identified in *P. papillatum*, *S. specki* and *N. karyoxenos* (figure 1; electronic supplementary material, figure S1*a*). Additionally, SarB was identified as a highly divergent orthologue in all diplonemids (electronic supplementary material, figure S1*a*). Duplication of Sec23 clearly took place in the euglenozoan ancestor (electronic supplementary material, figure S1*b*). Euglenozoan also possess both the LECA paralogues (Sec24I and Sec24II) of Sec24 with euglenids having duplicated Sec24I (figure 1; electronic supplementary material, figure S1*c*). Sec31 is present as a single paralogue, whereas Sec12 is missing in the entire euglenozoan clade (figure 1; electronic supplementary material, figure S1*d*). Although Sec16 was identified in *T. brucei*, *L. major* and *B. saltans*, we did not find orthologues in euglenids and diplonemids with these searches (but see below).

**Figure 2.**
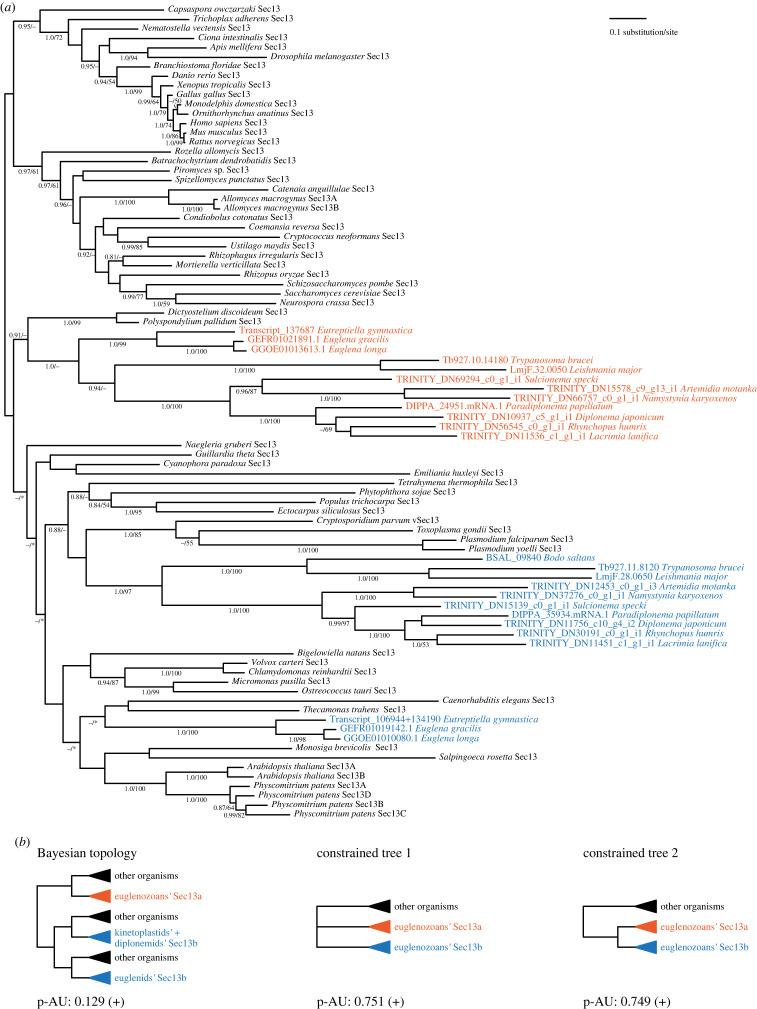
Phylogenetic analyses of Sec13. (*a*) MrBayes topology of Sec13 phylogenetic tree is shown, onto which posterior probabilities (PP) and bootstrap support (BS) values from RAxML are overlayed. Support values for PP < 0.8 and BS < 50% are denoted by a dash (–), whereas an asterisk (*) marks a topology that was not retrieved in a particular analysis. Sec13a paralogues are shown in orange, while Sec13b are shown in blue in all panels. (*b*) Alternative topologies constraining monophyly of euglenozoan Sec13 are shown together with *p*-value. A plus sign (+) indicates that the topology was not rejected by the AU test.

**Figure 3.**
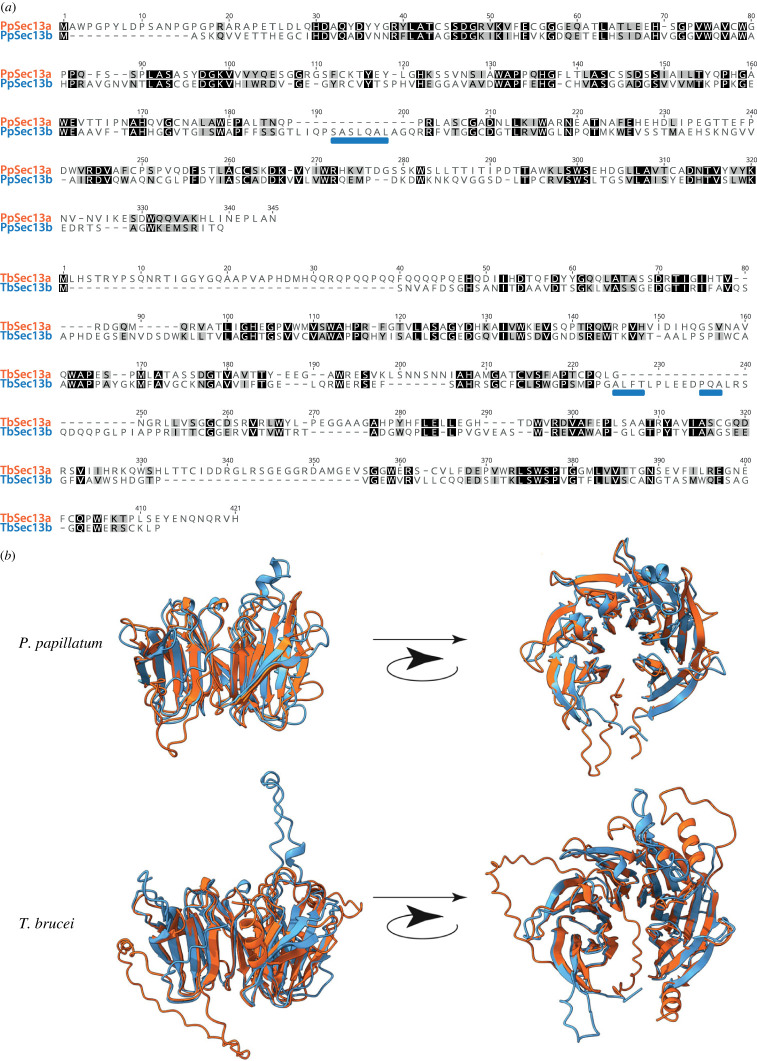
Structural divergence between Sec13a and Sec13b. (*a*) Alignments of Sec13 paralogues for *P. papillatum* and *T. brucei*. Please note that TbSec13a and TbSec13b are also named TbSec13.1 (accession number Tb927.10.14180) and TbSec13.2 (accession number Tb927.11.8120). (*b*) Overlain AlphaFold predictions for Sec13a and Sec13b (colours correspond to figure 1) for *P. papillatum* and *T. brucei* to illustrate the positions of indel peptide sequences (marked by blue rectangles in (*a*)). Note that the positions of indels are distinct, but probably provide significantly unique binding sites to discriminate between protein interactions and hence complex association.

Furthermore, all euglenozoans contain two paralogues of Sec13 (hereafter Sec13a and Sec13b) except for *B. saltans*, where only Sec13b was identified (figure 1). However, because of low statistical support in the backbone of the phylogenetic tree (figure 2*a*), we performed the approximately unbiased (AU) test constraining monophyly of all euglenozoan Sec13 paralogues, consistent with a hypothesis of duplication of Sec13 at the base of the euglenozoan clade. Indeed, this alternative topology was not rejected (figure 2*b*). Significantly, in *P. papillatum* the Sec13 paralogues share only 28% sequence similarity (figure 3*a*), even though they retain a predicted β-propeller architecture, *albeit* with clear regions of predicted structural divergence (figure 3*b*). We took advantage of the recently established genetic modification system [19,20] to investigate Sec13 functions in *P. papillatum*.

### 2.2. Gene tagging, protein–protein interactions and localization of PpSec13a

**Figure 4.**
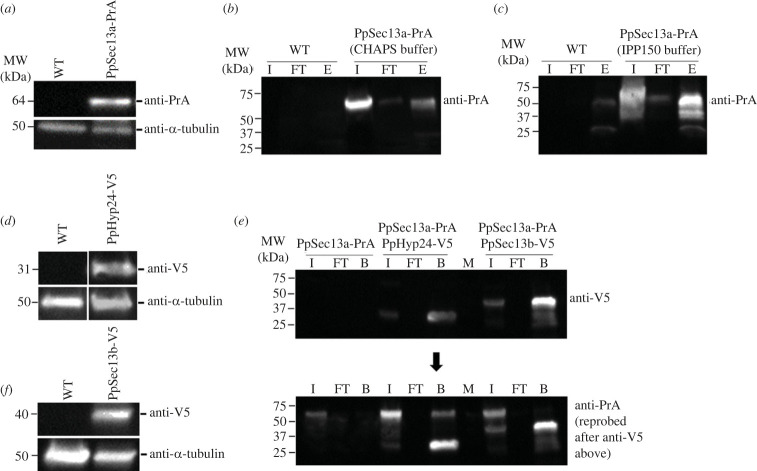
Immunoblot analyses and immunoprecipitations of PrA- and V5-tagged cell lines. Left panel: Immunoblots of *P. papillatum* wild type (WT) cell line along with tagged cell lines show the expression of tagged proteins (*a*) PpSec13a-PrA, (*d*) PpHyp24-V5 and (*f*) PpSec13b-V5. Rabbit anti-PrA (1 : 10 000) or mouse anti-V5 (1 : 2000) antibodies were used and mouse anti-α-tubulin (1 : 5000) was used as a loading control. Right panel: Immunoprecipitations of PrA- or V5- tagged cell lines to identify the interacting proteins of PpSec13a, PpHyp24 or PpSec13b were performed using the following conditions: (*b*) PpSec13a-PrA (CHAPS buffer)+IgG Sepharose beads; (*c*) PpSec13a-PrA (IPP150 buffer)+IgG Sepharose beads; (*e*) PpSec13a-PrA+PpHyp24-V5 (IPP150 buffer)+V5-trapped magnetic beads (middle part of the membranes); and PpSec13a-PrA+PpSec13b-V5 (IPP150 buffer)+V5-trapped magnetic beads (right part of the membranes). The presence of PpSec13b::V5 and PpHyp24::V5 in the bound fraction was verified by immunoblot using mouse anti-V5 (1 : 2000) antibody and reprobing the membrane with the anti-PrA antibody (1 : 10 000) showed also the presence of PpSec13a::PrA in the bound fraction of PpSec13a-PrA+PpHyp24-V5 cell line. PpSec13a::PrA cell line was used as a control. The triplicates of eluates (*b,c*) or beads (*e*) were analysed by mass spectrometry (figure 5). Wild-type (WT) strain or PpSec13a-PrA were used as controls. I, input; FT, flow-through; E, eluate; B, beads; M, marker. For more details see Material and Methods.

The PpSec13a gene of *P. papillatum* was tagged to identify protein-protein interactions and to determine intracellular localization. Plasmid pDP002 [19], containing a protein A (PrA) tag and neomycin as a selectable marker was used for endogenous tagging at the C-terminus (see Methods and electronic supplementary material, table S3). Expression of PrA-tagged PpSec13a was verified by immunoblotting (figure 4*a*).

**Figure 5.**
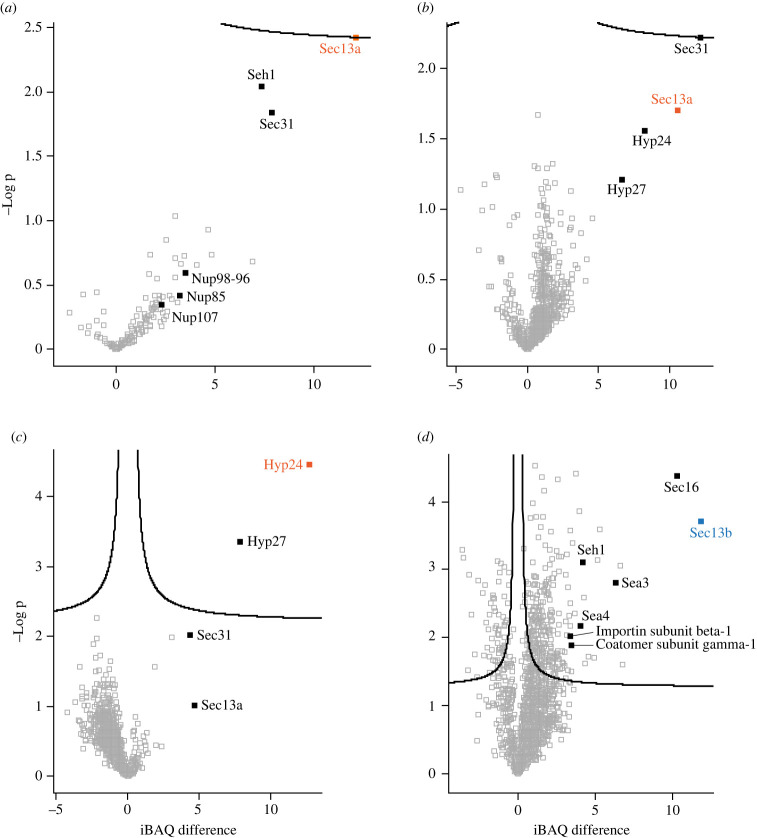
Volcano plot presentation of analysed proteins. The results of immunoprecipitations of PpSec13a-PrA (figure 4*b,c*), PpHyp24-V5 and PpSec13b-V5 (figure 4*e*) are shown as Volcano plots showing interacting proteins of (*a*) PpSec13a-PrA tagged protein (CHAPS buffer), (*b*) PpSec13a-PrA tagged protein (IPP150 buffer), (*c*) PpHyp24-V5 tagged protein in PpSec13a-PrA background and (*d*) PpSec13b-V5 tagged protein in PpSec13a-PrA background. The individual tagged proteins are shown in colours that correspond to colours in figure 1.

To purify PpSec13a together with interacting proteins, we performed immuno-isolations with PpSec13a::PrA-tagged cell lysates with parental/wild type (WT) cell lysates serving as a control. Since Sec13 plays a role in at least three distinct processes in animals, fungi and plants, we tested various immunoprecipitation conditions to attempt to preserve as many potential interactions as possible. Initially, we used a procedure with a buffer containing CHAPS as previously applied for immuno-isolation of the NPC from the related flagellate *T. brucei* [30]. Following verification by immunoblotting (figure 4*b*), liquid chromatography-tandem mass spectrometry (LC-MS/MS) identified in the purified immunoprecipitates four NPC components, namely PpNup107, PpNup98–96, PpNup85 and PpSeh1. These immunoprecipitates also contained an orthologue of Sec31, which again in animals, fungi and plants forms heterodimer with Sec13 to create the COPII coat (figure 5*a*; electronic supplementary material, figure S2*a*; electronic supplementary material, tables S4 and S5).

In a parallel strategy we isolated PpSec13a using an IPP150-containing buffer (see Material and Methods). Immunoblot analysis confirmed the presence of PpSec13a-PrA in the eluate fraction following immunoprecipitation (IP) (figure 4*c*). Subsequent MS analysis identified again PpSec31, as well as two hypothetical proteins with so far unknown function that we designate here, based on their predicted molecular weight, PpHyp24 (DIPPA_09665) and PpHyp27 (DIPPA_31723) (figure 5*b*; electronic supplementary material, figures S2*b,c* and table S4).

**Figure 6.**
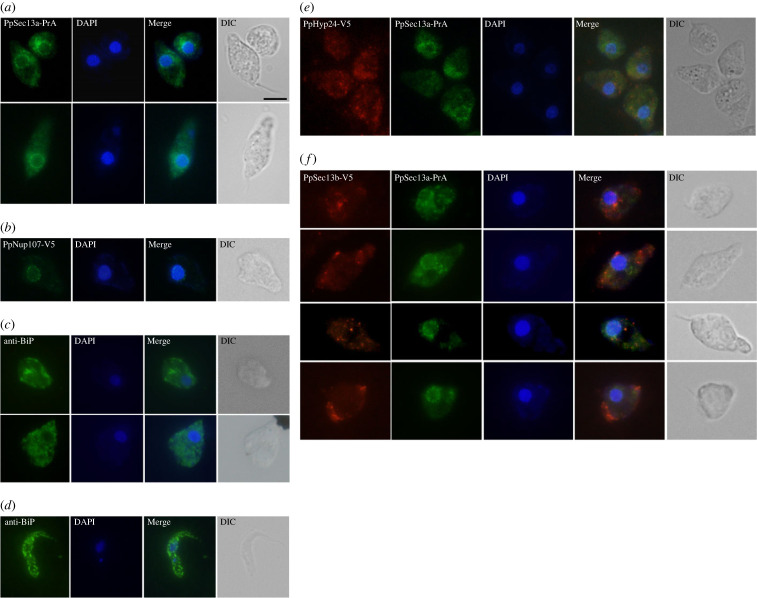
Localization and co-localization of PrA- and V5-tagged proteins in *P. papillatum*. (*a*) PpSec13a-PrA, (*b*) PpNup107-V5, (*e*) PpHyp24-V5 in PpSec13a-PrA background, and (*f*) PpSec13b-V5 in PpSec13a-PrA background tagged cell lines were analysed using immunofluorescence assay and stained with polyclonal anti-PrA antibody (green) or monoclonal anti-V5 antibody (red). To visualize the endoplasmic reticulum (ER) in *P. papillatum,* the wild-type cells were stained using anti-BiP antibody (*c*); *T. brucei* was used as a control (*d*). DNA was stained with DAPI (blue). DIC, Differential interference contrast. Scale bar: 5 µm.

Next, we used indirect immunofluorescence microscopy (IFA) to determine the location of PpSec13a using an anti-PrA antibody (figure 6*a*). This revealed a prominent signal around the nuclear envelope, verifying the interactions with NPC components, but also a punctate pattern, suggesting the presence of PpSec13a in the COPII complex, consistent with the identified interaction with PpSec31. The localization of PpNup107 (DIPPA_22976), an interacting partner of PpSec13a that we also tagged (electronic supplementary material, table S3), was consistent with a presence in the NPC (figure 6*b*).

In the absence of any IFA studies on *P. papillatum*, we decided to further investigate the punctate pattern by using an anti-BiP antibody (figure 6*c*), which serves as an ER marker in the related *T. brucei* (figure 6*d*). The observed punctate pattern is highly reminiscent of the ER signal in *T. brucei* (figure 6*c*,*d*), but could not be used for co-localization as both this and the anti-tag antibodies are anti-rabbit.

### 2.3. Tagging, interacting partners and localization of PpHyp24

To confirm the interaction between PpHyp24 and PpSec13a, we tagged the PpHyp24 gene in PpSec13a::PrA-tagged cell line to perform a reciprocal IP. Plasmid pDP011 was designed with a 3xV5 tag and the hygromycin B resistance gene (electronic supplementary material, figure S3) as a backbone for PCR amplification (electronic supplementary material, table S3) and electroporated into *P. papillatum*. The expression of V5-tagged PpHyp24 in PpSec13::PrA cells was verified by immunoblotting (figure 4*d*). Immunoprecipitates of PpHyp24-tagged cell lysates were prepared with anti-V5 magnetic beads and subjected to LC-MS/MS. The presence of PpHyp24::V5 in the bound fraction was verified by immunoblot and reprobing the membrane with the anti-PrA antibody showed also the presence of PpSec13a::PrA (figure 4*e*). LC-MS/MS of the IP revealed enrichment of all four proteins previously identified by PpSec13a::PrA immuno-isolation, validating the composition of this heterotetrameric complex (figure 5*c*; electronic supplementary material, table S4).

Next, we used indirect immunofluorescence microscopy to determine the localization of PpHyp24. It revealed a punctate distribution of the target protein, only partially overlapping with PpSec13a (figure 6*e*), which is consistent with the immunoprecipitation result.

### 2.4. Tagging, interacting partners and localization of PpSec13b

To identify protein interacting partners of the second Sec13 paralogue, PpSec13b, we again used the pDP011 plasmid for tagging. Similarly, as with the PpHyp24::V5 cell line, we established a cell line expressing both PpSec13b::V5 and PpSec13a::PrA. Expression of PpSec13b::V5 was confirmed by immunoblot analysis (figure 4*f*).

Immunoprecipitation using anti-V5 magnetic beads also failed to identify a PpSec13a and PpSec13b interaction (figure 4*e*), but LC-MS/MS analysis of the PpSec13b IP revealed enrichment of three gene products, DIPPA_10282, DIPPA_06252 and DIPPA_21857, in addition to the affinity handle (figure 5*d*; electronic supplementary material, table S4). DIPPA_10282 is a Sec16 orthologue and, using DIPPA_10282 as query, we identified orthologues in additional diplonemid (electronic supplementary material, table S2) and euglenid transcriptomes; by phylogenetics these sequences form a robust clade with the kinetoplastid Sec16 orthologues (electronic supplementary material, figure S1*e*). Given the identification of PpSec16, this prompted us to revisit the distribution of Sec16 across eukaryotes, identifying orthologues in the slime mold *Fonticula alba*, the red alga *Cyanidioschyzon merolae*, the apicomplexan parasites *Cryptosporidium parvum*, *Babesia bovis* and *Theileria equi*, and multiple haptophytes (electronic supplementary material, table S6), indicating that Sec16 is present more broadly than previous evidence suggested. BLAST searches of DIPPA_21857 and orthologues from other euglenozoans revealed DIPPA_21857 as orthologous to Sea4. However, Sea2, Sea3 and Sea4 are themselves paralogues and BLAST searches of DIPPA_06252 and euglenozoan orthologues identified these proteins as components of the SEA/GATOR complex but without sufficient E-value differences to discriminate between Sea2, Sea3 or Sea4 (electronic supplementary material, table S7). Moreover, we identified an additional Sea2 gene in the genomes and transcriptomes of euglenozoans (DIPPA_06078 in *P. papillatum*) (figure 1; electronic supplementary material, table S7), suggesting that DIPPA_06252 (and the euglenozoan orthologues) represents Sea3.

Phylogenetic analyses of euglenozoan and selected eukaryote sequences clearly distinguishes Sea2, Sea3 and Sea4 clades (electronic supplementary material, figure S2*d*), but paralogues of the kinetoplastid Sea2 and Sea3 were not clustered within any of these clades. We therefore used the kinetoplastid candidates as queries in reverse BLASTs against *P. papillatum*. In nearly every case this confirmed the initial annotations as Sea2 and Sea3 (electronic supplementary material, table S7). In animals and fungi, Sea2, Sea3 and Sea4 are components of the SEA/GATOR complex and regulate the TORC signalling pathway involved in controlling cell growth, stress responses and other processes.

Finally, we determined the localization of both PpSec13a and PpSec13b by performing double labelling immunofluorescence analysis using anti-PrA and anti-V5 antibodies, respectively (figure 6*f*). In agreement with the IP data, the intracellular localizations of PpSec13a and PpSec13b are distinct, and fully consistent with the divergent protein–protein interaction cohort identified for each paralogue.

### 2.5. Endocytosis and ultrastructure of *P. papillatum*

**Figure 7.**
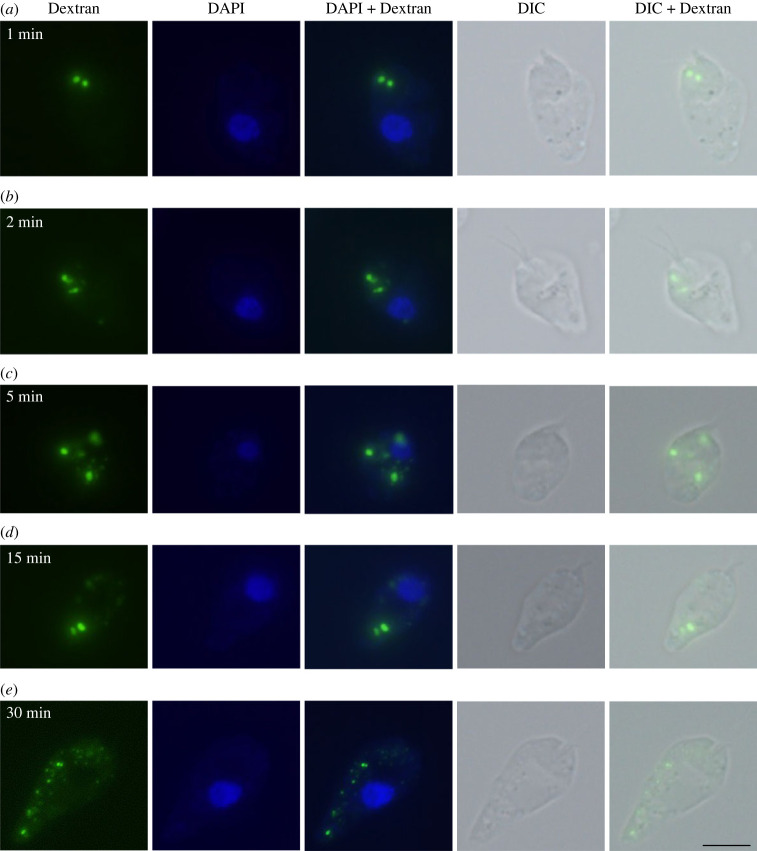
Endocytosis of *P. papillatum* monitored by fluorescent dextran. Cells were preincubated in artificial sea water without nutrients for 15 min before the addition of 5 mg ml^−1^ FITC-labelled dextran. Cells were incubated with dextran for (*a*) 1 min, (*b*) 2 min, (*c*) 5 min, (*d*) 15 min, and (*e*) 30 min, subsequently fixed with 4% paraformaldehyde, mounted on slides with ProlongGold antifade reagent with DAPI and observed using fluorescence microscopy. Scale bar: 5 µm.

**Figure 8.**
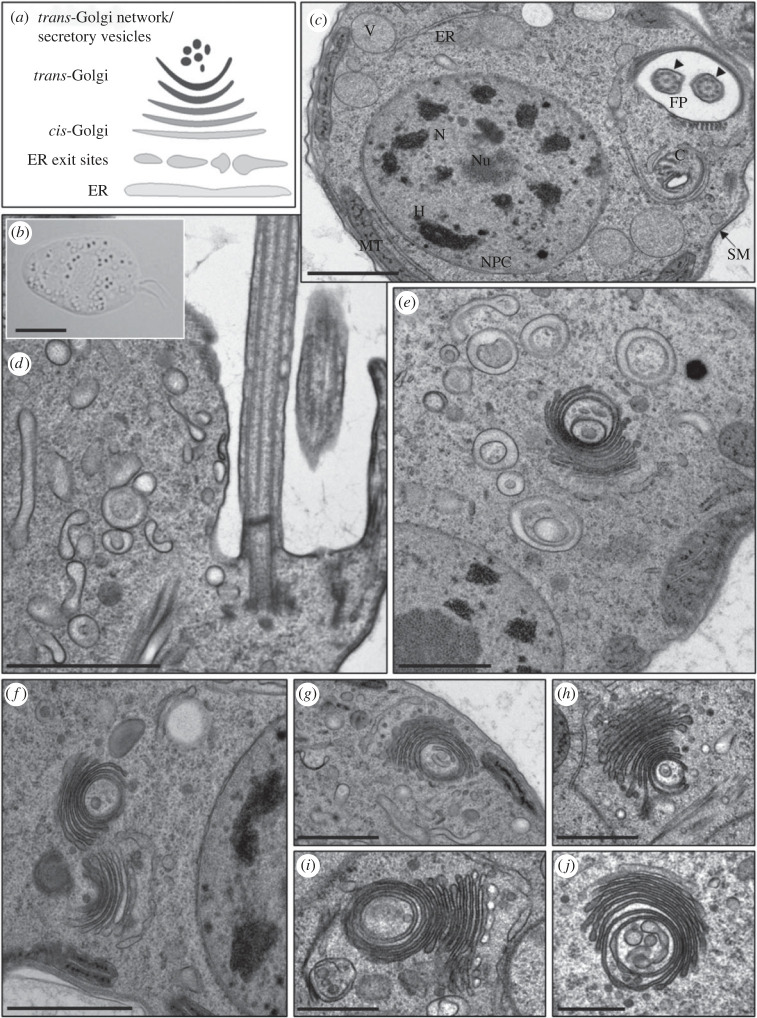
Transmission electron microscopy of *P. papillatum*. (*a*) Schematic depiction of ER-Golgi structure. (*b*) Light microscopy of cultured cell, showing abundant cytoplasmic vesicles. (*c*) Cross section of the cell showing the following cellular structures and organelles: C, cytostome; ER, endoplasmic reticulum; FP, flagellar pocket; H, heterochromatin; MT, mitochondrion; N, nucleus; Nu, nucleolus; NPC, nuclear pore complex; SM, subpelicullar microtubules; V, undetermined vesicle; arrowheads indicate two cross-sectioned flagella within the flagellar pocket. (*d*) Longitudinally sectioned flagellar pocket with flagella and numerous vesicles. (*e–j*) Details of prominent Golgi complexes. Scale bars: (*b*) 5 µm; (*c–f*) 1 µm; (*g–j*) 500 nm.

To better understand the organization of the diplonemid endomembrane system and the distribution of PpSec13 paralogues, we used fluorescent (FITC) dextran to monitor endocytosis by light microscopy (figure 7) and also imaged the *P. papillatum* cells by transmission electron microscopy (figure 8). Dextran has been successfully used to monitor uptake of material by fluid phase endocytosis in many organisms, including the kinetoplastid *T. brucei*.

Diplonemids showed an uptake of FITC dextran already after 1 minute-long incubation (figure 7*a*), followed by extensive consumption, which was monitored for 2 to 15 min (figure 7*b–d*). The decreasing and fragmented signal indicates ongoing digestion that was observed after 30 min into the experiment (figure 7*e*).

To observe endocytic pathway at the ultrastructural level, we subjected *P. papillatum* (figure 8*b*) to transmission electron microscopy, which allowed the identification of the flagellar pocket, cytopharynx, subpellicular microtubules, terminal endosomes/lysosomes, a large nucleus with prominent nuclear pores and NPCs, nucleolus and regions of heterochromatin (figure 8*c,d*). Moreover, we observed a complex series of abundant membrane-bound structures, which includes the ER, visualized as tubular vesicles of varying length associated with the Golgi complex (figure 8*a*, *e–j*). Endosome-like structures, manifested frequently as vesicles within vesicles, may represent either true endosomes, multivesicular bodies or autophagosomes, possible locations for TORC. We also noted that the Golgi complex is particularly prominent and highly coordinated with the ER and ER exit sites (ERES). This diplonemid Golgi complex is highly distinctive, with an extremely concave morphology that includes circular *trans*-most cisternal profiles and which are associated with vesicular structures also harbouring internal membrane profiles. Significantly, the *cis*-Golgi cisternae are associated with a putative ER tubule, most likely the location of ERES and hence COPII. As the cisternae progress from the *cis* to *trans* faces, their content becomes increasingly electron dense, reflecting concentration of cargo for export to the cell surface. In some sections (figure 8*d–j*) we observe putative transport vesicles of approximately 10 nm diameter associated with the Golgi complex and of similar electron density to the *trans*-cisternal compartments, which we propose represent vesicles destined for the surface.

The morphology clearly demonstrates the presence of a highly organized ERES, a stacked Golgi complex and the NPCs. The occasional presence of more than one Golgi complex in the sections (figure 8*f*) is most likely consistent with the specific cells being in mitosis. The organization of the anterograde pathway is quite striking with a clear high order organization between the ERES and the *cis*-face of the Golgi complex. Moreover, all of the expected compartments and transport events predicted by affinity isolation are supported by the presence of the relevant organelles. Specifically, a stacked Golgi complex (Sec16), ERES (Sec31), endosomal degradative compartments (SEA/GATOR) and the NPC are all clear and present, also consistent with the contributions of PpSec13 paralogues as observed at the light level; specifically punctate nuclear rim staining and cytoplasmic puncta that likely correspond to the ERES, possible Golgi association and endosomes. Furthermore, the highly organized architecture of the *P. papillatum* cell may also suggest a significant level of precision with respect to regulation and organellar morphology in these protists.

## 3. Discussion

Eukaryogenesis began over a billion years ago culminating in a highly complex last eukaryotic common ancestor, but the evolutionary and diversification processes underpinning this event remain ongoing within extant lineages [31–33]. Part of the molecular machinery facilitating the evolution of multiple compartments arose through expansions of paralogue families [1,34]. Here we demonstrate that Sec13, recognized as a component of at least three protocoatomer complexes, achieved unique diversity within the Euglenozoa, an early diverging lineage [19], with two Sec13 paralogues, each of which exhibits distinct sub-functionalization. This conclusion is supported by interactome proteomics and subcellular localization using epitope-tagged gene products.

The Sec13a paralogue has been analysed quite extensively in *T. brucei* (also named TbSec13.1; accession number Tb927.10.14180), demonstrated as a component of the NPC, localized into the ERES [35]. Similarly, TbSec13a possesses a nuclear localization signal, while TbSec13b (also named TbSec13.2; accession number Tb927.11.8120) prominently lacks one, which suggests that TbSec13b is unlikely to be an NPC component. In *T. brucei* immunoisolation of TbSec13a does not capture TbSec16 or the SEA/GATOR complex components, suggesting a similar division of labour between the two paralogues as in *P. papillatum* [30]. Localization of both TbSec13a and 13b as part of the Tryptag database appear identical and highlight likely Golgi and endosomal compartments, consistent with the expected localization for TbSec13b, but with no evidence for nuclear rim location. We suggest that this is likely an artefact; TbSec13a is a clear NPC component and has been localized to the NPC [35], and the TrypTag dataset were not validated for faithful insertion onto the intended coding sequence. Consistently, in *P. papillatum* we identified PpSec16 and Sea proteins only from immuno-isolations of PpSec13b but not PpSec13a, suggesting a conserved distinction between Sec13a and Sec13b across the Euglenozoa.

The arrangement and coordination of the ERES and NPC transport is clearly divergent in trypanosomes and related flagellates. The lineage is characterized by a cell surface dominated by lipid-anchored proteins and glycoconjugates. The two paralogues of Sec23 and Sec24 form distinct heterodimers of TbSec23b/TbSec24a and TbSec23a/TbSec24b (TbSec24a corresponds to the LECA Sec24I and TbSec24b to the LECA Sec24II) and, importantly, TbSec23b/TbSec24a specifically mediates anterograde transport of the GPIanchored proteins [36], indicating a complex mechanism regulating the ER export. (Please note that TbSec23a, TbSec23b, TbSec24a, and TbSec24b are also named TbSec23.1, accession number Tb927.8.3660; TbSec23.2, accession number Tb927.10.7740; TbSec24.1, accession number Tb927.3.1210; and TbSec24.2, accession number Tb927.3.5420, respectively.) Furthermore, Sec12, the guanine nucleotide exchange factor for Sar1 in yeasts and animals, is absent [37], while there is a considerable heterogeneity in the Sar1/SarB paralogue cohort across the Euglenozoa in general. TbSec31 is regulated by the cell cycle-dependent kinase CDK1 [38] and, significantly, both replication of the Golgi complex and the ERES is highly coordinated with entry to the G_1_ phase of the cell cycle. Finally, direct interaction between Sec13 and Sec16 indicates a role for the latter in both anterograde transport, as well as in the maintenance of Golgi morphology [39], and given the highly organized *P. papillatum* Golgi complex, we speculate that Sec16 may have a similar role in this protist. Indeed, ultrastructurally very prominent Golgi complexes have been described in other diplonemids, namely *R. humris*, *L. lanifica*, *S. specki*, *Flectonema neradi* [40] and *N. karyoxenos* [41]. It is worth to mention here that based on recent environmental DNA sequencing, diplonemids represent the most diverse and 5^th^ most abundant marine protists [18], which shows their enormous success in the extant oceans.

The conservation of two Sec13 paralogues across the Euglenozoa indicates a probably fundamental requirement for distinct control mechanisms of cellular functions within this lineage. Association of PpSec13a and TbSec13a with the NPC and COPII indicates a role in intracellular transport, while the presence of PpSec13b and TbSec13b in the respective SEA/GATOR complexes as well as with the autophagy-associated Sec16, suggests a closer integration with pathways sensitive to nutrient status for the latter paralogue, and in particular amino acid levels. This may indicate a requirement to uncouple nuclear and ER transport processes from changes to amino acid levels, but the original requirement may have been subsequently lost. Divergence of the Sec13 paralogues, together with coevolution of their interacting partners, likely provides a lock preventing the loss of either Sec13 paralogue.

## 4. Material and methods

### 4.1. Sequence searches, structure predictions and phylogenetic analyses

COPII subunits were retrieved by BLAST+v2.8.1 [42] searches using discobid sequences [8] as queries in the genome of *Paradiplonema papillatum* [21], transcriptomes of diplonemids *Diplonema japonicum*, *Rhynchopus humris*, *Lacrimia lanifica*, *Sulcionema specki*, *Artemidia motanka* and *Namystynia karyoxenos* [22], genome of the kinetoplastid *Bodo saltans* [43], and transcriptomes of the euglenids *Euglena longa* [44] and *Eutreptiella gymnastica* [45] (reassembly available at https://doi.org/10.5281/zenodo.257410) [46]. To identify more divergent sequences, more sensitive searches by HMMER v3.3 based on profile hidden Markov models [47] were performed in parallel. Protein domains were predicted by InterProScan [48] implemented in Geneious v2020.2.5 [49]. Secondary structures of proteins were de novo predicted by AlphaFold2 [50], visualized and overlayed by ChimeraX v1.4 [51].

Datasets of COPII components were obtained from previous studies [8,9,35]. As PpHyp27 did not retrieve any orthologues in reverse BLAST searches against NCBI, both PpHyp24 and PpHyp27 were subjected to more sensitive profile searches using HHBlits [52] and best hits served as datasets for phylogenetic analyses. Found sequences were added to the datasets, aligned by MAFFT v7.458 under L-INS-i strategy [53], and poorly aligned positions were removed by trimAl using -gt 0.8 option [54]. Maximum-likelihood (ML) phylogenetic analysis was performed by RAxML v8.2.8 [55] under the LG4X mixture model using 100 rapid bootstrap replicates (-f a). Bayesian inference was performed by MrBayes v3.2.7a [56] under a mixed amino acid model, with at least 10 million Markov chain Monte Carlo generations and 4 gamma rate categories. Sampling frequency was set to every 1000 generations with the first 25% of the runs discarded as burn-in. Bootstrap support values were overlaid onto the MrBayes tree topology with posterior probabilities.

To test the monophyly of euglenozoan Sec13 sequences, the AU test was performed. We constructed ML trees using IQ-TREE v2.2.0 [57] under the LG+C20+F+G model constraining the monophyly of euglenozoan Sec13 using -g option. The AU test was run in IQ-TREE on the constrained trees, the topology retrieved from the Bayesian analysis, and 1,000 distinct local topologies saved during ML analysis. Topologies that returned an AU-test *p*-value < 0.05 were rejected.

### 4.2. Strain and cultivation of *P. papillatum*

*P. papillatum* (ATCC 50162) was cultivated axenically at 27°C in an artificial sea salt medium as described previously [20] and cell density was measured manually using the Neubauer cell chamber.

### 4.3. Endogenous C-terminal gene tagging and used cell lines

All cassettes were designed and prepared by fusion PCR approach using Phusion polymerase (NEB Biolabs, M0530S) as described elsewhere [20]. For PrA tagging, PrA + Neo^R^ cassette was amplified from pDP002 plasmid, while for V5 tagging, 3xV5 + Hyg^R^ cassette was amplified from the newly designed pDP011 plasmid (electronic supplementary material, figure S3). Used primers and PCR product sizes are listed in electronic supplementary material, table S3. The gel-purified cassettes were ethanol-precipitated and sequentially electroporated into the *P. papillatum* cells [20,58]. For transformation, a total of 5 × 10^7^ cells were harvested and electroporated with appropriate DNA constructs (cassettes; see below) as described previously [20].

Eighteen to 24 h after electroporation, transfectants were subjected to selection in 24 well plates at 27°C with increasing concentrations of either G418 (25–80 µg ml^−1^) for establishing the PpSec13a-PrA cell line, or hygromycin (100–225 µg ml^−1^) for creating the PpNUP107-V5 cell line, or both (G418 and hygromycin) at the same time for establishing the PpSec13a-PrA + PpHyp24-V5 and PpSec13a-PrA + PpSec13b-V5 double-tagged cell line. After two to three weeks, successful transfectants were obtained and each clone was expanded to a volume of 20 ml. The expression and verification of expected size of the tagged proteins in obtained resistant cell lines was verified by immunoblot analysis.

In this study we selected and further used the following cell lines: 1) PpSec13a-PrA cell line growing in media supplemented with 67.5 µg ml^−1^ G418; 2) PpSec13a-PrA+PpHyp24-V5 and 3) PpSec13a-PrA+PpSec13b-V5 both growing in media supplemented with 67.5 µg ml^−1^ G418 and 100 µg ml^−1^ of hygromycin and 4) PpNup107-V5 growing in media supplemented with 125 µg ml^−1^ of hygromycin.

### 4.4. Immunoblot analysis

Immunoblot (western blot) analysis of *P. papillatum* samples was performed as described previously [20]. Membranes were first incubated with rabbit anti-PrA (1 : 10 000; Sigma-Aldrich, P3775) or mouse anti-V5 (1:2000; ThermoFisher Scientific, 37–7500) primary antibody at 4°C overnight or at room temperature for 2 h. After five washes in phosphate buffered saline supplemented with Tween (PBS-T; 0.05% (v/v) Tween in 1 × PBS), membranes were incubated with HRP-coupled anti-rabbit (1 : 1000; Sigma-Aldrich, A21428) or anti-mouse (1 : 1000; Sigma-Aldrich, A9044) secondary antibody at room temperature for 1 h. After five PBS-T washes, the signal was developed using Clarity Western ECL Substrate (Bio-Rad). The mouse anti-α-tubulin antibody (1:10 000; Sigma-Aldrich, T9026) was used as a loading control.

### 4.5. Immunoprecipitation

For PrA-tagged cell line, 5 × 10^8^ cells (PpSec13a-PrA) were grown at 27°C in media with selection antibiotics (see above). Cells were harvested at 1000 g for 10 min and subsequently resuspended either in 1 ml ice-cold IPP150 buffer (10 mM Tris-HCl pH 6.8, 150 mM NaCl, 0.1% IGEPAL CA-630; Sigma I8896) or in CHAPS buffer (20 mM HEPES pH 7.4, 100 mM NaCl, 0.5% CHAPS; Roche 10810118001), both supplemented with 1 × complete EDTA-free protease inhibitors (Sigma, 11873580001) and five times passed through a 30-gauge needle. The lysate was subsequently cleared twice by centrifugation (12 000 g, 10 min, 4°C) and the supernatant was incubated with 75 µl of IgG Sepharose 6 Fastbeads (GE Healthcare, 52-2083-00 AH) by rotating at 4°C for 2 to 3 h to enable binding of the tagged protein. The beads were washed five times using the same buffer as for cell lysis. The complex of PpSec13a-PrA together with its potential interaction partners were eluted using 100 µl of 0.1 M glycine (pH 3.0) by rotating for 5 min at room temperature and immediately neutralized with 10 µl of 1 M Tris-HCl (pH 9.0). Aliquots of input, flow through and elution fractions were processed for immunoblotting. The elution fraction was subsequently sent for MS analysis.

For V5-tagged cell lines, 5 × 10^8^ cells (PpSec13a-PrA+PpHyp24-V5 or PpSec13a-PrA+PpSec13b-V5) were grown at 27°C in media with appropriate selection antibiotics. Cells were harvested at 1000 g for 10 min, resuspended in 1 ml ice-cold IPP150 buffer and subsequently processed using similar protocol as above with the following exceptions: 1) supernatant was incubated with 50 µl of V5-Trap magnetic Particles M-270 (Chromotek, v5td-20; the advantage of these beads is that there are no heavy and light antibody chains present in the bound fraction and therefore even proteins of 25/50 kDa can be seen on immunoblot), 2) last two washes of beads were done using a buffer without detergent and the beads were sent for MS analysis. For all IP experiments, three replicates of each sample were processed by MS. Wild-type cells were used as a control.

### 4.6. Mass spectrometry and data analysis of immunoprecipitated samples

Trypsin-digestion of the eluted PrA-tagged bait and wild-type controls or the V5-paramagnetic beads incubated with V5-tagged bait and wild-type controls was performed prior to LC-MS/MS as follows. IP samples were resuspended in 100 mM TEAB containing 2% SDC, and cysteines were reduced with 10 mM final concentration of TCEP and blocked with 40 mM final concentration of chloroacetamide (60°C for 30 min). Samples were cleaved on beads with 1 µg of trypsin at 37°C overnight. After digestion, samples were centrifuged, and supernatants were collected and acidified with TFA to 1% final concentration. SDC was removed by extraction with ethylacetate, and peptides were desalted using in-house made stage tips packed with C18 discs (Empore). Nano Reversed phase column (EASY-Spray column, 50 cm × 75 µm ID, PepMap C18, 2 µm particles, 100 Å pore size) was used for LC/MS analysis. Mobile phase buffer A was composed of water and 0.1% formic acid, and mobile phase B was composed of acetonitrile and 0.1% formic acid. Samples were loaded onto the trap column (Acclaim PepMap300, C18, 5 µm, 300 Å Wide Pore, 300 µm × 5 mm, 5 Cartridges) for 4 min at 15 µl min^−1^. Loading buffer was composed of water, 2% acetonitrile and 0.1% trifluoroacetic acid. Peptides were eluted with Mobile phase B gradient from 4% to 35% B in 60 min. Eluting peptide cations were converted to gas-phase ions by electrospray ionization and analysed on a Thermo Orbitrap Fusion (Q-OT- qIT, Thermo). Survey scans of peptide precursors from 350 to 1400 *m/z* were performed at 120 K resolution (at 200 *m/z*) with a 5 × 10^5^ ion count target. Tandem MS was performed by isolation at 1,5 Th with the quadrupole, HCD fragmentation with normalized collision energy of 30, and rapid scan MS analysis in the ion trap. The MS/MS ion count target was set to 104 and the max injection time was 35 ms. Only those precursors with charge state 2–6 were sampled for MS/MS. The dynamic exclusion duration was set to 45 s with a 10 ppm tolerance around the selected precursor and its isotopes. Monoisotopic precursor selection was turned on and the instrument was run in top speed mode with 2 s cycles.

Data were processed using MaxQuant v1.6.14, which incorporates the Andromeda search engine [59]. A custom protein sequence database of *P. papillatum* proteins (43 871 sequences) supplemented with frequently observed contaminants was used to identify proteins. Search parameters were the default ones employed by MaxQuant for Orbitrap analysers with full trypsin specificity and allowing for up to two missed cleavages. Carbamidomethylation of cysteine was set as a fixed modification and oxidation of methionine and N-terminal protein acetylation were allowed as variable modifications. Match between runs for biological replicates was part of the experimental design. Peptides were required to be at least seven amino acids long, with false discovery rates of 0.01 calculated at the levels of peptides, proteins, and modification sites based on the number of hits against the reversed sequence database. iBAQ indices (raw intensities divided by the number of theoretical peptides) were used for protein quantification, which allows comparing of protein abundances both within samples and between them. After filtering to remove any proteins with less than two unique peptides, an Andromeda score of less than 20 and less than two valid values in the respective bait replicates, the obtained data were processed in Perseus v1.6.14 as described previously [60].

### 4.7. Immunofluorescence assay

Twenty to 30 ml of a log phase culture was centrifuged at 1000 g for 5 min in order to visualize localization of PpSec13a, PpHyp24, PpSec13b and PpNup107 in *P. papillatum*. Cells were resuspended in 500 µl of 4% paraformaldehyde (dissolved in sea water) and fixed for 20 min on Superfrost plus slides (Thermo Scientific, J1800AMNZ) at room temperature. The fixative was washed out from cells with 1 × PBS. For antibody staining, cells were permeabilized in ice-cold methanol for 20 min. The slides were kept in a humid chamber throughout the procedure. Afterwards, the slides were washed with 1 × PBS, and blocked for 45 min in 5.5% (w/v) fetal bovine serum in PBS-T. The blocking solution was removed, and cells were washed with 1 × PBS. Rabbit anti-PrA (1 : 2000; Sigma, P3775) and/or mouse anti-V5 (1 : 150; ThermoFisher Scientific, 37–7500) primary antibody diluted in 3% (w/v) bovine serum albumin (Sigma, A4503) in PBS-T was added on slides and incubated either for 2 h at room temperature or at 4°C overnight, covered with parafilm. Next, the primary antibody was removed, and slides were washed three times with PBS-T and twice with 1 × PBS. AlexaFluor488-labelled goat anti-rabbit (1 : 1000; Invitrogen, A11034) and/or AlexaFluor555-labelled goat anti-mouse (1 : 1000; Invitrogen, A21422) secondary antibody was added and incubated at room temperature for 1 h in the dark, covered with parafilm. All slides were then rinsed three times with PBS-T and twice with 1 × PBS and coated with 4',6-diamidino-2-phenylindole (DAPI) containing the antifade reagent ProlongGold (Life Technologies). Similarly, rabbit anti-BiP antibody (1 : 10 000; gift from James D. Bangs) was used to visualize the endoplasmic reticulum (ER) in *P. papillatum*, *T. brucei* was used as a control. Images were acquired using an Olympus BX63 automated fluorescence microscope equipped with an Olympus DP74 digital camera and evaluated with cellSens Dimension software (Olympus).

### 4.8. Fluorescent dextran staining

Fluorescent (FITC) dextran (Sigma Aldrich, FD500S) was used to monitor endocytosis of *P. papillatum* cells. One ml of exponentially growing culture (1−2 × 10^6^ cells ml^−1^) was preincubated for 15 min in artificial sea water (36 g l^−1^ sea salts; Sigma, S9883) without nutrition to starve the cells before the addition of 5 mg ml^−1^ FITC-labelled dextran. Incubations continued for 1 to 30 min, cells were then fixed in 4% paraformaldehyde, washed with 1 × PBS, mounted on slides with Prolong Gold antifade reagent with DAPI and observed using fluorescence microscopy.

### 4.9. Transmission electron microscopy

Transmission electron microscopy samples were prepared by high pressure freezing technique (HPF), a very rapid method that prevents the formation of ice crystals that could damage the cells ultrastructure. Briefly, 5 × 10^8^ *P. papillatum* cells were concentrated by centrifugation and processed as described previously [61]. Ultrathin sections were cut using an ultramicrotome (Leica Microsystems) and collected on copper grids, which were contrasted in ethanolic uranyl acetate and lead citrate and observed using a JEOL 1010 microscope at accelerating voltage of 80 kV. Images were captured with an Olympus Mega View III camera.

## Data Availability

The DNA sequence of pDP011 was deposited in GenBank under the OQ547858 accession number. The mass spectrometry proteomics data have been deposited to the ProteomeXchange Consortium via the PRIDE [62] partner repository with the dataset identifier PXD037122. The data are provided in electronic supplementary material [63].

## References

[1] Dacks JB, Field MC. 2007 Evolution of the eukaryotic membrane-trafficking system: Origins, tempo and mode. J. Cell Sci. **120**, 2977-2985. (10.1242/jcs.013250) 17715154

[2] Wideman JG, Muñoz-Gómez SA. 2016 The evolution of ERMIONE in mitochondrial biogenesis and lipid homeostasis: an evolutionary view from comparative cell biology. Biochim. Biophys. Acta—Mol. Cell Biol. Lipids **1861**, 900-912. (10.1016/j.bbalip.2016.01.015) 26825688

[3] Dacks JB, Field MC. 2018 Evolutionary origins and specialisation of membrane transport. Curr. Opin. Cell Biol. **53**, 70-76. (10.1016/j.ceb.2018.06.001) 29929066 PMC6141808

[4] Rutherford S, Moore I. 2002 The *Arabidopsis* Rab GTPase family: another enigma variation. Curr. Opin. Plant Biol. **5**, 518-528. (10.1016/S1369-5266(02)00307-2) 12393015

[5] Carlton JM et al. 2007 Draft genome sequence of the sexually transmitted pathogen *Trichomonas vaginalis*. Science **315**, 207-212. (10.1126/science.1132894) 17218520 PMC2080659

[6] Neumann N, Lundin D, Poole AM. 2010 Comparative genomic evidence for a complete nuclear pore complex in the last eukaryotic common ancestor. PLoS ONE **5**, e13241. (10.1371/journal.pone.0013241) 20949036 PMC2951903

[7] Rout MP, Field MC. 2022 Coatomer in the universe of cellular complexity. Mol. Biol. Cell **33,** pe8. (10.1091/mbc.e19-01-0012) 36399624 PMC9727805

[8] Schlacht A, Dacks JB. 2015 Unexpected ancient paralogs and an evolutionary model for the COPII coat complex. Genome Biol. Evol. **7**, 1098-1109. (10.1093/gbe/evv045) 25747251 PMC4419792

[9] Vargová R, Wideman JG, Derelle R, Klimeš V, Kahn RA, Dacks JB, Eliáš M. 2021 A eukaryote-wide perspective on the diversity and evolution of the ARF GTPase protein family. Genome Biol. Evol. **13**, evab157. (10.1093/gbe/evab157) 34247240 PMC8358228

[10] Tang BL. 2017 Sec16 in conventional and unconventional exocytosis: working at the interface of membrane traffic and secretory autophagy? J. Cell. Physiol. **232**, 3234-3243. (10.1002/jcp.25842) 28160489

[11] Yorimitsu T, Sato K. 2020 Sec16 function in ER export and autophagy is independent of its phosphorylation in *Saccharomyces cerevisiae*. Mol. Biol. Cell **31**, 149-156. (10.1091/mbc.E19-08-0477) 31851588 PMC7001475

[12] Fontoura BMA, Blobel G, Matunis MJ. 1999 A conserved biogenesis pathway for nucleoporins: proteolytic processing of a 186-kilodalton precursor generates Nup98 and the novel nucleoporin, Nup96. J. Cell Biol. **144**, 1097-1112. (10.1083/jcb.144.6.1097) 10087256 PMC2150585

[13] Dokudovskaya S et al. 2011 A conserved coatomer-related complex containing Sec13 and Seh1 dynamically associates with the vacuole in *Saccharomyces cerevisiae*. Mol. Cell. Proteomics **10**, M110.006478. (10.1074/mcp.M110.006478) PMC3108837 21454883

[14] Loissell-Baltazar YA, Dokudovskaya S. 2021 SEA and GATOR 10 years later. Cells **10**, 2689. (10.3390/cells10102689) 34685669 PMC8534245

[15] Barlowe C et al. 1994 COPII: a membrane coat formed by Sec proteins that drive vesicle budding from the endoplasmic reticulum. Cell **77**, 895-907. (10.1016/0092-8674(94)90138-4) 8004676

[16] Flegontova O, Flegontov P, Londoño PAC, Walczowski W, Šantić D, Edgcomb VP, Lukeš J, Horák A. 2020 Environmental determinants of the distribution of planktonic diplonemids and kinetoplastids in the oceans. Environ. Microbiol. **22**, 4014-4031. (10.1111/1462-2920.15190) 32779301

[17] Prokopchuk G, Korytář T, Juricová V, Majstorović J, Horák A, Šimek K, Lukeš J. 2022 Trophic flexibility of marine diplonemids—switching from osmotrophy to bacterivory. ISME J. **16**, 1409-1419. (10.1038/s41396-022-01192-0) 35042972 PMC9039065

[18] Tashyreva D et al. 2022 Diplonemids—a review on ‘new’ flagellates on the oceanic block. Protist **173**, 125868. (10.1016/j.protis.2022.125868) 35339983

[19] Faktorová D et al. 2020 Genetic tool development in marine protists: emerging model organisms for experimental cell biology. Nat. Methods **17**, 481-494. (10.1038/s41592-020-0796-x) 32251396 PMC7200600

[20] Faktorová D, Kaur B, Valach M, Graf L, Benz C, Burger G, Lukeš J. 2020 Targeted integration by homologous recombination enables *in situ* tagging and replacement of genes in the marine microeukaryote *Diplonema papillatum*. Environ. Microbiol. **22**, 3660-3670. (10.1111/1462-2920.15130) 32548939

[21] Valach M et al. 2023 Recent expansion of metabolic versatility in *Diplonema papillatum*, the model species of a highly speciose group of marine eukaryotes. BMC Biol. **21**, 99. (10.1186/s12915-023-01563-9) 37143068 PMC10161547

[22] Kaur B, Záhonová K, Valach M, Faktorová D, Prokopchuk G, Burger G, Lukeš J. 2020 Gene fragmentation and RNA editing without borders: eccentric mitochondrial genomes of diplonemids. Nucleic Acids Res. **48**, 2694-2708. (10.1093/nar/gkz1215) 31919519 PMC7049700

[23] Berriman M et al. 2005 The genome of the African trypanosome *Trypanosoma brucei*. Science **309**, 416-422. (10.1126/science.1112642) 16020726

[24] Ivens AC et al. 2005 The genome of the kinetoplastid parasite, *Leishmania major*. Science **309**, 436-442. (10.1126/science.1112680) 16020728 PMC1470643

[25] Flegontov P et al. 2016 Genome of Leptomonas pyrrhocoris: a high-quality reference for monoxenous trypanosomatids and new insights into evolution of Leishmania. Sci. Rep. **6**, 23704. (10.1038/srep23704) 27021793 PMC4810370

[26] Ebenezer TE et al. 2019 Transcriptome, proteome and draft genome of *Euglena gracilis*. BMC Biol. **17**, 11. (10.1186/s12915-019-0626-8) 30732613 PMC6366073

[27] Sevova ES, Bangs JD. 2009 Streamlined architecture and glycosylphosphatidylinositol-dependent trafficking in the early secretory pathway of African trypanosomes. Mol. Biol. Cell **20**, 4739-4750. (10.1091/mbc.E09-07-0542) 19759175 PMC2777104

[28] Kruzel EK, Zimmett GP, Bangs JD. 2017 Life stage-specific cargo receptors facilitate glycosylphosphatidylinositol-anchored surface coat protein transport in *Trypanosoma brucei*. mSphere **2**, e00282-17. (10.1128/msphere.00282-17) 28713858 PMC5506558

[29] Demmel L, Melak M, Kotisch H, Fendos J, Reipert S, Warren G. 2011 Differential selection of golgi proteins by COPII Sec24 isoforms in procyclic *Trypanosoma brucei*. Traffic **12**, 1575-1591. (10.1111/j.1600-0854.2011.01257.x) 21801288

[30] Obado SO, Brillantes M, Uryu K, Zhang W, Ketaren NE, Chait BT, Field MC, Rout MP. 2016 Interactome mapping reveals the evolutionary history of the nuclear pore complex. PLoS Biol. **14**, e1002365. (10.1371/journal.pbio.1002365) 26891179 PMC4758718

[31] Roger AJ, Susko E, Leger MM. 2021 Evolution: Reconstructing the timeline of eukaryogenesis. Curr. Biol. **31**, R193-R196. (10.1016/j.cub.2020.12.035) 33621507

[32] Eme L, Spang A, Lombard J, Stairs CW, Ettema TJG. 2017 Archaea and the origin of eukaryotes. Nat. Rev. Microbiol. **15**, 711-723. (10.1038/nrmicro.2017.133) 29123225

[33] Koonin EV. 2015 Origin of eukaryotes from within archaea, archaeal eukaryome and bursts of gene gain: eukaryogenesis just made easier? Phil. Trans. R. Soc. B **370**, 20140333. (10.1098/rstb.2014.0333) 26323764 PMC4571572

[34] Devos D, Dokudovskaya S, Alber F, Williams R, Chait BT, Sali A, Rout MP. 2004 Components of coated vesicles and nuclear pore complexes share a common molecular architecture. PLoS Biol. **2**, e380. (10.1371/journal.pbio.0020380) 15523559 PMC524472

[35] DeGrasse JA, Dubois KN, Devos D, Siegel TN, Sali A, Field MC, Rout MP, Chait BT. 2009 Evidence for a shared nuclear pore complex architecture that is conserved from the last common eukaryotic ancestor. Mol. Cell. Proteomics **8**, 2119-2130. (10.1074/mcp.M900038-MCP200) 19525551 PMC2742445

[36] Sevova ES, Bangs JD. 2009 Streamlined architecture and glycosylphosphatidylinositol-dependent trafficking in the early secretory pathway of African trypanosomes. Mol. Biol. Cell. **20**, 4739-4750. (10.1091/mbc.e09-07-0542) 19759175 PMC2777104

[37] Wang YN, Wang M, Field MC. 2010 *Trypanosoma brucei*: Trypanosome-specific endoplasmic reticulum proteins involved in variant surface glycoprotein expression. Exp. Parasitol. **125**, 208-221. (10.1016/j.exppara.2010.01.015) 20109450 PMC2877885

[38] Hu H, Gourguechon S, Wang CC, Li Z. 2016 The G_1_ cyclin-dependent kinase CRK1 in *Trypanosoma brucei* regulates anterograde protein transport by phosphorylating the COPII subunit Sec31. J. Biol. Chem. **291**, 15 527-15 539. (10.1074/jbc.M116.715185) PMC4957039 27252375

[39] Sealey-Cardona M, Schmidt K, Demmel L, Hirschmugl T, Gesell T, Dong G, Warren G. 2014 Sec16 determines the size and functioning of the Golgi in the protist parasite, *Trypanosoma brucei*. Traffic **15**, 613-629. (10.1111/tra.12170) 24612401

[40] Tashyreva D et al. 2018 Phylogeny and morphology of new diplonemids from Japan. Protist **169**, 158-179. (10.1016/j.protis.2018.02.001) 29604574

[41] Prokopchuk G, Tashyreva D, Yabuki A, Horák A, Masařová P, Lukeš J. 2019 Morphological, ultrastructural, motility and evolutionary characterization of two new Hemistasiidae species. Protist **170**, 259-282. (10.1016/j.protis.2019.04.001) 31154071

[42] Altschul SF, Gish W, Miller W, Myers EW, Lipman DJ. 1990 Basic local alignment search tool. J. Mol. Biol. **215**, 403-410. (10.1016/S0022-2836(05)80360-2) 2231712

[43] Jackson AP et al. 2016 Kinetoplastid phylogenomics reveals the evolutionary innovations associated with the origins of parasitism. Curr. Biol. **26**, 161-172. (10.1016/j.cub.2015.11.055) 26725202 PMC4728078

[44] Záhonová K et al. 2018 Peculiar features of the plastids of the colourless alga *Euglena longa* and photosynthetic euglenophytes unveiled by transcriptome analyses. Sci. Rep. **8**, 17012. (10.1038/s41598-018-35389-1) 30451959 PMC6242988

[45] Keeling PJ et al. 2014 The Marine Microbial Eukaryote Transcriptome Sequencing Project (MMETSP): illuminating the functional diversity of eukaryotic life in the oceans through transcriptome sequencing. PLoS Biol. **12**, e1001889. (10.1371/journal.pbio.1001889) 24959919 PMC4068987

[46] Johnson LK, Alexander H, Brown CT. 2017 (all datasets) MMETSP re-assemblies [Data set]. *Zenodo*. (10.5281/zenodo.257410)

[47] Eddy SR. 2009 A new generation of homology search tools based on probabilistic inference. Genome Inf. **23**, 205-211. (10.1142/9781848165632_0019) 20180275

[48] Jones P et al. 2014 InterProScan 5: genome-scale protein function classification. Bioinformatics **30**, 1236-1240. (10.1093/bioinformatics/btu031) 24451626 PMC3998142

[49] Kearse M et al. 2012 Geneious Basic: an integrated and extendable desktop software platform for the organization and analysis of sequence data. Bioinformatics **28**, 1647-1649. (10.1093/bioinformatics/bts199) 22543367 PMC3371832

[50] Jumper J et al. 2021 Highly accurate protein structure prediction with AlphaFold. Nature **596**, 583-589. (10.1038/s41586-021-03819-2) 34265844 PMC8371605

[51] Pettersen EF, Goddard TD, Huang CC, Meng EC, Couch GS, Croll TI, Morris JH, Ferrin TE. 2021 UCSF ChimeraX: Structure visualization for researchers, educators, and developers. Protein Sci. **30**, 70-82. (10.1002/pro.3943) 32881101 PMC7737788

[52] Remmert M, Biegert A, Hauser A, Söding J. 2012 HHblits: Lightning-fast iterative protein sequence searching by HMM-HMM alignment. Nat. Methods **9**, 173-175. (10.1038/nmeth.1818) 22198341

[53] Katoh K, Standley DM. 2013 MAFFT multiple sequence alignment software version 7: improvements in performance and usability. Mol. Biol. Evol. **30**, 772-780. (10.1093/molbev/mst010) 23329690 PMC3603318

[54] Capella-Gutiérrez S, Silla-Martínez JM, Gabaldón T. 2009 trimAl: a tool for automated alignment trimming in large-scale phylogenetic analyses. Bioinformatics **25**, 1972-1973. (10.1093/bioinformatics/btp348) 19505945 PMC2712344

[55] Stamatakis A. 2014 RAxML version 8: A tool for phylogenetic analysis and post-analysis of large phylogenies. Bioinformatics **30**, 1312-1313. (10.1093/bioinformatics/btu033) 24451623 PMC3998144

[56] Ronquist F et al. 2012 MrBayes 3.2: efficient Bayesian phylogenetic inference and model choice across a large model space. Syst Biol **61**, 539-542. (10.1093/sysbio/sys029) 22357727 PMC3329765

[57] Minh BQ, Schmidt HA, Chernomor O, Schrempf D, Woodhams MD, Von Haeseler A, Lanfear R, Teeling E. 2020 IQ-TREE 2: new models and efficient methods for phylogenetic inference in the genomic era. Mol. Biol. Evol. **37**, 1530-1534. (10.1093/molbev/msaa015) 32011700 PMC7182206

[58] Kaur B, Valach M, Peña-Diaz P, Moreira S, Keeling PJ, Burger G, Lukeš J, Faktorová D. 2018 Transformation of *Diplonema papillatum*, the type species of the highly diverse and abundant marine microeukaryotes Diplonemida (Euglenozoa). Environ. Microbiol. **20**, 1030-1040. (10.1111/1462-2920.14041) 29318727

[59] Cox J, Neuhauser N, Michalski A, Scheltema RA, Olsen JV, Mann M. 2011 Andromeda: A peptide search engine integrated into the MaxQuant environment. J. Proteome Res. **10**, 1794-1805. (10.1021/pr101065j) 21254760

[60] Zoltner M et al. 2020 Suramin exposure alters cellular metabolism and mitochondrial energy production in African trypanosomes. J. Biol. Chem. **295**, 8331-8347. (10.1074/jbc.RA120.012355) 32354742 PMC7294092

[61] Yurchenko V, Votỳpka J, Tesařová M, Klepetková H, Kraeva N, Jirku M, Lukeš J. 2014 Ultrastructure and molecular phylogeny of four new species of monoxenous trypanosomatids from flies (Diptera: Brachycera) with redefinition of the genus *Wallaceina*. Folia Parasitol. **61**, 97-112. (10.14411/fp.2014.023) 24822316

[62] Perez-Riverol Y et al. 2022 The PRIDE database resources in 2022: A hub for mass spectrometry-based proteomics evidences. Nucleic Acids Res. **50**, D543-D552. (10.1093/nar/gkab1038) 34723319 PMC8728295

[63] Faktorová D, Záhonová K, Benz C, Dacks JB, Field MC, Lukeš J. 2023 Functional differentiation of Sec13 paralogues in the euglenozoan protists. Figshare. (10.6084/m9.figshare.c.6673618) PMC10264103 37311539

